# The role of big data management, data registries, and machine learning algorithms for optimizing safe definitive surgery in trauma: a review

**DOI:** 10.1186/s13037-024-00404-0

**Published:** 2024-06-20

**Authors:** Hans-Christoph Pape, Adam J. Starr, Boyko Gueorguiev, Guido A. Wanner

**Affiliations:** 1 Department of Trauma Surgery, University Hospital of Zurich, Raemistr. 100, Zurich, 8091 Switzerland; 2 Department of Orthopaedic Surgery, Parkland Memorial Hospital, University of Texas Southwestern, 4900 Harry Hines Blvd, Dallas, TX 75235 USA; 3 AO Research Institute Davos, Clavadelerstr. 8, Davos, 7270 Switzerland; 4 Department of Spine & Trauma Surgery, Bethanien Hospital, Toberlstr. 51, Zurich, 8044 Switzerland

**Keywords:** Polytrauma, Trauma registry, Artificial intelligence, Big Data, Safe definitive surgery, Outcome prediction

## Abstract

Digital data processing has revolutionized medical documentation and enabled the aggregation of patient data across hospitals. Initiatives such as those from the AO Foundation about fracture treatment (AO Sammelstudie, 1986), the Major Trauma Outcome Study (MTOS) about survival, and the Trauma Audit and Research Network (TARN) pioneered multi-hospital data collection. Large trauma registries, like the German Trauma Registry (TR-DGU) helped improve evidence levels but were still constrained by predefined data sets and limited physiological parameters. The improvement in the understanding of pathophysiological reactions substantiated that decision making about fracture care led to development of patient’s tailored dynamic approaches like the Safe Definitive Surgery algorithm. In the future, artificial intelligence (AI) may provide further steps by potentially transforming fracture recognition and/or outcome prediction. The evolution towards flexible decision making and AI-driven innovations may be of further help. The current manuscript summarizes the development of big data from local databases and subsequent trauma registries to AI-based algorithms, such as Parkland Trauma Mortality Index and the IBM Watson Pathway Explorer.

## Introduction

Along with the availability of hospital administration systems, the options for big data management have substantially improved [1]. Modern patient documentation systems are usually able to combine general clinical information that covers the hospital course, laboratory data and other sets, such as core radiological data [2]. Moreover, some recent systems include the option of developing apps to describe clinical syndromes and further diagnostic aids [3, 4].

Registries have frequently been generated alongside certification processes and have provided large volumes of information from multiple hospitals of a given country. The range of their given data sets is usually limited, as they focus on certain clinically relevant aspects (registries for trauma, geriatric trauma, or other diseases). Thereby, registry-based data bases frequently provide unidirectional information and can be used only for their given purpose [5, 6]. Along these lines, the most expanded sets of data are mostly generated by insurance companies [7]. Some of them combined the results after trauma or other diseases to assess a prognosis of the medical treatment result [8]. Others primarily focus on selecting quality programs and some are even developed to cover issues of reimbursement [9].

Prior to the development of registries, physicians were involved in several manners, and some focused on the results of their given treatments. In this line, the AO Foundation was the first institution collecting data sets that go beyond the purpose of their development. The first so-called “femur fracture collection study” summarized data from 1127 patients with femur shaft fractures treated with intramedullary nails. Although this study was designed to document healing issues after intramedullary fixation, their results demonstrated an unusually high rate of pulmonary complications – namely ARDS and pulmonary embolism – in young patients after isolated femur fractures. This data set triggered the discussion about the fracture fixation influence on the development of complications. Of note, this discussion initiated a large number of publications about 10 years before the first article from the major trauma outcome study and 15 years before the initiation of the concept for damage control orthopaedics [10].

Others are designed to provide data for patient assessment and teaching purposes or to develop guidelines and management tools for future use [11]. Furthermore, assessment of the quality of care following secondary referral could be performed recently [12]. Also, evaluations on rare injury combinations and their management have been performed [13] even within the frame of international comparisons [14]. Finally, quality assessment in terms of surgeon`s experiences and outcomes has been performed.

Another new approach generating big imaging data sets has been suggested to improve the quality of surgical studies that have been proved as biased by the skills level of the performing surgeons [15]. Complete intraoperative documentation of surgical procedures is disseminated in a standardized way. A subsequent access and electronic management of data sets online have been suggested by a group of experienced surgeons [16], allowing for post hoc analysis by artificial intelligence (AI) [17]. Moreover, recent progress in biochemical and molecular biological analytics is known as a provider of big data sets for characterization of the genomic-, transcriptomic-, and metabolomic patients’ status. When combined with clinical data, these translational approaches are intended to improve the prognostic fidelity, allowing for individual risk stratification of trauma patients and safe definitive surgery decisions [18, 19].

The current research article summarizes the development of options for generation of big data in the clinical setting of trauma patients. More specifically, it provides an analysis of the most pertinent data sets utilized for trauma patients. Moreover, it compares the options among registries and local data sets and analyzes the role of the data sets for the concept development of safe definitive surgeries in trauma patients. Finally, it emphasizes a new approach for surgical quality control (ICUC), scores that were generated based on the initiatives listed above, and a brief outlook on future AI options [20].

## Methods

### Inclusion criteria

- Big data were included if they were generated from more than 500 patients, if the individual numbers were limited, or if the combination of multiple time points and parameters in a given data set exceeded 50,000.
- Systematic literature review.

1) Studies that investigate registry data and data exceeding the ability to be covered by one facility only;

2) Studies providing reliable information assessing diagnostic tools, including scoring systems, to facilitate profiling of injuries;

3) Studies that provide information to assess injury and fracture management. Case reports, defined as studies reporting data on a sample size of less than 5 patients were excluded. Results from meeting abstracts were also excluded.

The approach to generate the data was two-fold:

First, **a systematic literature** review was performed by the authors, using Medline database with the language selection being English or German. The authors focused on certain eligibility criteria for multiply injured patients as indicated before. Original articles were included if published between Jan 1, 1985 and May 15, 2023. No language restrictions were applied. Data sources included MEDLINE, Embase, and the Web of Science. Disagreements were resolved in discussion.

Second, an **expert analysis** has been generated on the concepts of known data analysis options. All authors are experts in the field of data analysis. The expert analysis was then combined with the predefined data interpretation to describe the way experienced clinicians react to the technical progress in data management.

The authors have had involvement in this manuscript as follows:

**AS** is an expert in development of a hospital index to monitor the risk of mortality (Parkland mortality index) [21]. Moreover, he is the developer in a frame to facilitation reduction for pelvic fractures ( [22] Starr frame).

**BG** is vice director of the AO Research Institute Davos, Switzerland. He is also in charge of the Biomedical Development program at the Institute and has been involved in multiple studies with a multi-center design.

**GAW** has been a leader in the Network of the German Trauma System, where he chaired a cross-border trauma network as organizer and referral hospital (Schwarzwald-Baar Hospital, Level-1 trauma Center). In terms of generating data on a hospital level, he has generated a data base to develop data on posttraumatic complications [23]. Moreover, he has recently developed a new method to perform intraoperative visualization and documentation in pelvic surgery [24] based on mixed reality technology.

**HCP** is a founding member of the German Trauma Registry and was involved in the development of scores and concepts, which were defined on the basis of a big data set. Among these are the Thoracic trauma score (TTS) [25], the evidence based Definition of Polytrauma [26], the damage control concept [27], and the Safe Definitive Surgery concept [28].

### Definitions

*Big data* were defined as digitally available data, as described in the inclusion criteria.

The *Injury Severity Score* was used to determine injury severity.

The *Glasgow Coma Scale* [29] was utilized to assess head injuries, if available.

For definition of *Multiple organ failure* (MOF), the Sequential Organ Failure Assessment score was used (SOFA), or other scores, as indicated in the given manuscript [30].

*Sepsis* was diagnosed in patients having three or more points in a specific organ with at least two organs failing at the same time.

*In hospital mortality* was defined as the passing of a patient during the treatment in the primary care hospital.

### Inclusion criteria

Studies were included if they overlooked data from more than 100 patients from a single institution or 300 from a multicenter data base, if they were generated by registries, or if their size was large enough to develop a new clinical concept or a definition of a disease.

### Exclusion criteria

Studies were excluded if their results were not reconfirmed after publication, or if they were inconclusive.

### Distribution of data collections

- *local distribution* was defined as data collection on a hospital level.
- *regional distribution* was defined as data collection among several hospitals.
- *country wide distribution* was defined as data collection within the borders of a given country.
- *registry based distribution* was defined as data collection within a defined registry, independent of regions or borders.

Data collection processes were further divided into those that are determined to develop general patient data, or those on local injury severity, to predict the hospital course (complications, sepsis), to predict general outcome after trauma, or to develop scoring systems.

### Artificial narrow intelligence (narrow AI; ANI)

Narrow AI (ANI) is AI programmed to perform a single task (e.g. to play chess). ANI systems can attend a task in real-time but pulling information from a specific data set. ANI systems process data and complete tasks at a significantly quicker pace than any human being can. The main purpose of it is to enable humans to improve overall productivity, efficiency, and quality of life. ANI systems, such as IBM’s Watson, for example, is able to harness the power of AI to assist doctors to make data-driven decisions [2].

### Artificial general intelligence (strong AI; AGI)

Artificial General Intelligence (AGI) or “strong” AI refers to machines that exhibit human intelligence. AGI can successfully perform intellectual tasks that are covered by a human being. This sort of AI that can be seen in movies such as “Her” (where humans interact with machines and operating systems that are conscious, sentient, and driven by emotion).

### Artificial super intelligence (super AI; ASI)

Artificial Super Intelligence (ASI) is defined as “any intellect that greatly exceeds the cognitive performance of humans in virtually all domains of interest” (Nick Bostrom). This condition surpasses human intelligence in all aspects — such as creativity, general wisdom, and problem-solving [31].

## Results

The first larger database in trauma patients that exceeds the numbers defined above has been the AO collection of patients with femur fractures (Table Ia), published in 1986, i.e. about a decade before the MTOS was generated. The initial purpose of this data base has been to document the healing process in patients who sustained isolated femur fractures. The AO has subsequently performed multiple studies that include multiple centers and has focused on different topics, as indicated in Table Ia. All these studies have been generated, funded and developed by the AO with no external funding. It was then followed by multiple other clinical studies and the FROST initiative, which is about to be completed. It summarizes all patients with tibial fractures regardless of the type of fixation. Likewise, the periprosthetic fracture registry documents patients with these kind of geriatric injuries (Table 1a).

**Table 1a:** Examples for studies initiated by the AO to determine outcome after certain fractures

Name	Region	Year of initiation and purpose of data collection
“AO Sammelstudie”	Europe	1980, outcome after femur fracture
AO “FROST Study”	Europe	2015, outcome after tibia fracture
AO “PPFx Study”	Europe	2015, outcome after periprosthetic fracture

The Major Trauma Outcome Study (MTOS) [32] summarized information on trauma patients (Table 1b). It collected data from hospitals in the USA and its results proved to be helpful for several reasons. On one hand, they served help to develop criteria to identify those hospitals where trauma patients were to be concentrated. On the other hand, it helped develop new scores to assess patients early and reduce issues that occurred when using the Injury severity score based on AIS (AIS/ISS). As it was developed to score patients with life threatening injuries, it became evident that by using the maximum AIS of a single body region only, a certain subset of patients was underdiagnosed, namely those with multiple extremity fractures [33]. This was the main reason why the New ISS (NISS) was developed [34].

**Table 1b:** Principles of quality control and trauma registries and quality control tools

Name	Region	Purpose of the registry
MTOS	USA	Assess outcome and develop quality control
TARN	GB	Quality control and trauma care audit
TR DGU	EU/ME	Quality control and research
Japanese Trauma Registry	Japan	Quality control and research

On a separate note, it was thought that physiological data should be added. This has lead to the development of a “Severity Characterization of Trauma” (ASCOT) [35], which provided a physiologic and anatomic characterization of injury severity. This score combined emergency department admission values of Glasgow Coma Scale, systolic blood pressure, respiratory rate, patient age, and AIS-85 anatomic injury scores to minimize the shortcomings of ISS [35].

Independent of the issues described above, the ISS has enabled authorities to describe and monitor different levels of hospitals and focus on those that require a larger volume and associated overhead costs. These regional distributions were associated with sharing of data between several hospitals, or hospital systems – they lead to the development of trauma systems and their associated certifications [36].

Associated with these developments was a subsequent country wide distribution. In the USA, the National Trauma Database was strictly based in the country of development [37]. In Europe, Great Britain developed the Trauma Audit and Research Network [38] and the network originated in Germany [39], which was the first to document across borders, i.e. in Belgium, Netherlands, Switzerland, and other countries including outside Europe (Table 1b).

These registry based distributions lead to distinct sets of data frequently used for quality control and assessment of mortality rates. Moreover, they have been used during a consensus process during the development of a definition for polytrauma patients. In this consensus process, a data set of more than 28,000 patients was used to test a hypothesis generated upon a suggestion developed by experts [26] (Table 2). Table 2 lists scoring systems developed to address clinical problems [40]. The underlying data bases are also documented [41]. As these have been described in various publications, we hereby refrain from describing them in detail [42–44].

**Table 2:** Development of scoring systems to address clinical problems

Name	Publication	Name of Score	Author, year
Abdominal trauma grading	Local data base	Moore Score	Moore, 1993
ARDS consensus definition	Delphi process	Berlin Definition	Bernard, 1993
Grading of Chest trauma	Local data base	TTS Score	Pape, 2000
Definition of polytrauma	Trauma Registry	Berlin Definition	Pape, 2014
Prediction tool for complications	Local Data base	Watson analytics	Mica, 2020
Data warehouse project	Local Data Base	Data warehouse	Niggli, 2021

Table 3 summarizes surgical tools for decision making in fracture care. The principles of surgical management of major fractures in multiple injured patients are described and set in relation to available parameters and data size [42, 45]. The use of databases can also be seen in the development of the fracture care of major fractures, where a development trend was seen towards the use of multiple parameters at multiple time points after admission in order to achieve the best possible patient safety. Subsequently, it was able to calculate threshold levels for distinct parameters, which were able to separate mortality rates and other clinical outcome parameters [43] (Table 3).

**Table 3:** Surgical tools for decision making in fracture care*

Reference, year	Pat. No	Type	Data base character	Principle	Endpoints / Parameter(s)
Bone, 1989	128	prosp. rand., single center	development data set	ETC	ventilation time
Goris, 1990	89	retrospective	development data set	ETC	ventilation time
Pape, 2005	-	retrospective, level IV	Local data base	DCO	multiple parameters
Nahm, 2012	750	retrospective, single center	development data set	EAC	admission lactate > 4 mmol/L
Dienstknecht, 2014	167	prospective, multi center	development data set	DCO	Acute lung failure, ALI
Hildebrand, 2015	23,210	retrospective, single center	development data set	DCO	Poly Trauma Grading Score
Halva, 2020	3668	retrospective, single center		SDS	4 different pathogen. cycles

* Principles of surgical management of major fractures in multiply injured patients in relation to available parameters and data size

**Fig. 1:**
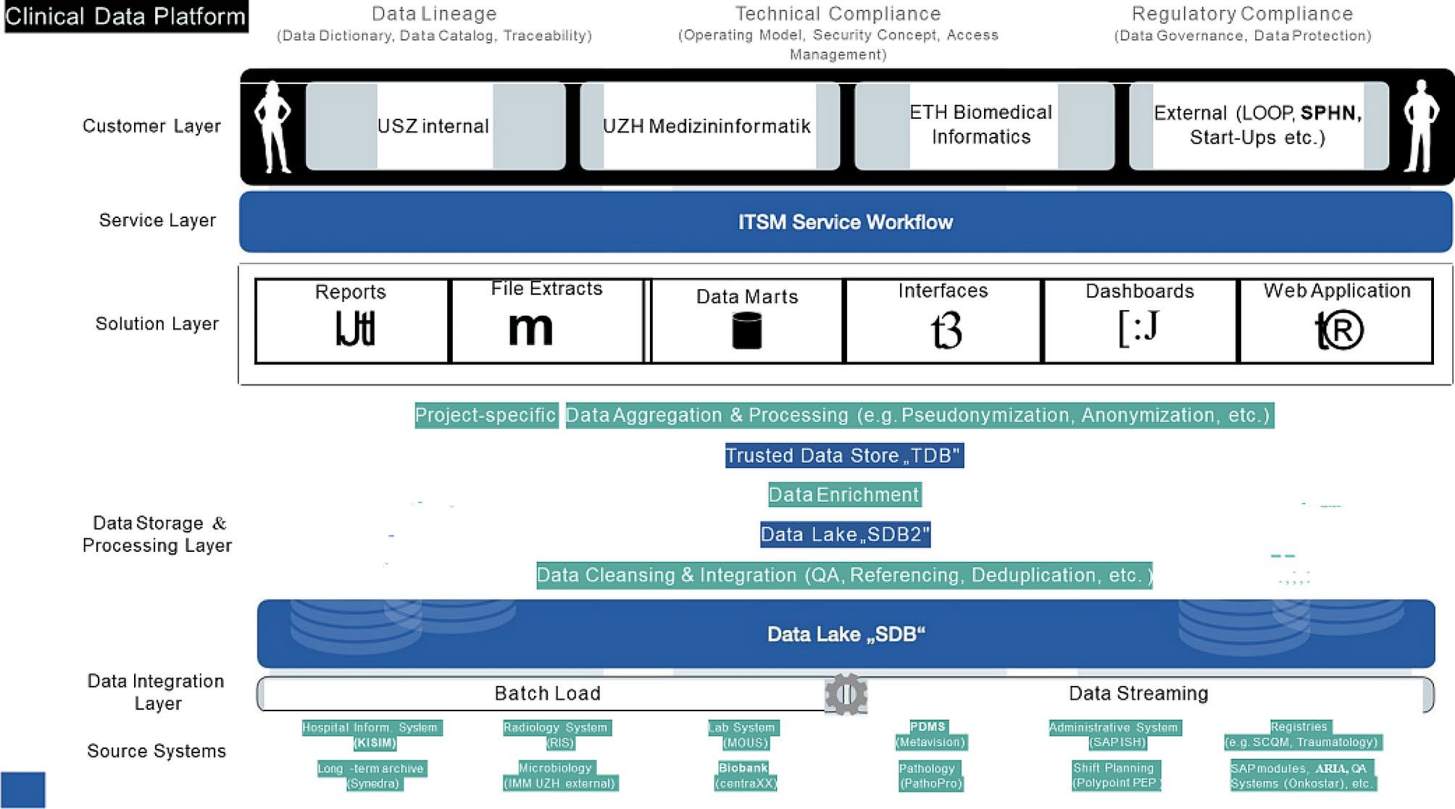
Documents how data can be derived from a hospital system to be available for clinicians, researchers, and data analyses (unpublished data)

Figure 1. Direct access to the online patient chart to combine patient data on a local level can be achieved by data warehouse and requires constant data exchange (unpublished data from Zurich University hospital, mentioned in Niggli et al., 2021 ().

**Fig. 2:**
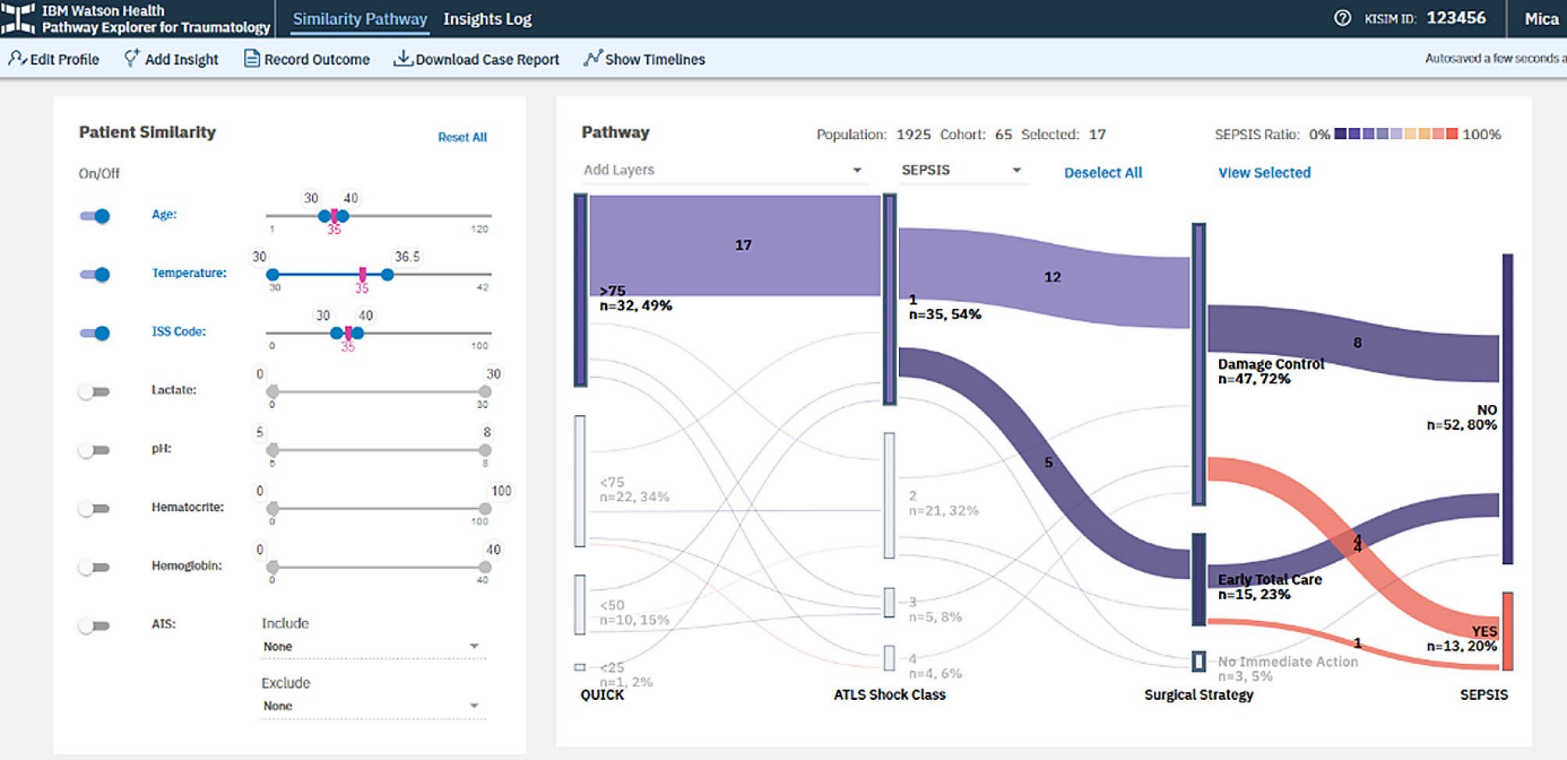
Example for the IBM Watson Health TRAUMA explorer, developed according to a data base of 3650 patients. The IBM Watson Health TRAUMA explorer can have access to the hospital data base system. Explanation: Level A: On the left side, the patient status can be assessed and the background of underlying patients can be selected (geriatric versus young). Then the surgical tactic can be selected and the system will provide information about the outcome seen in the selected patient group. Level B: The underlying patient population can be changed according to the expectations, such as age range, ISS range etc. This will determine the initial data of the pathway. Level C (visualization and Sankey diagram): According to different treatment options (resuscitation, surgical strategy etc.), the outcome can vary. Currently, the options for endpoints have been set to mortality, Sepsis and SIRS

Figure 2 depicts the Sankey visualization tool named IBM Watson. It utilizes acute data extracted from the hospital information system. However, it is not collected to verify a certain parameter. Instead, it utilizes the data to perform a visualization of the expected hospital course. This approach has been derived from the development of turbines, where a developer used pressure and flow diagnostics to improve the thrust of turbines of various shapes. This Sankey diagram can be used in various fields of medicine. Figure 2 shows a Sankey projection that was based on (about) 10 clinical laboratory values, and is able to determine a risk scenario based on a previous large group of more than 1000 patients with polytrauma as a comparison [1] (Fig. 2). It projects the possible clinical course in the determinants listed under “pathway”, where coagulation, ATLS Shock severity, surgical strategy and outcome are determined. Although it is not justified to use these data to predict outcome, it may serve as a valuable tool to mimic clinical scenarios for physicians in training and interdisciplinary groups [46].

**Fig. 3:**
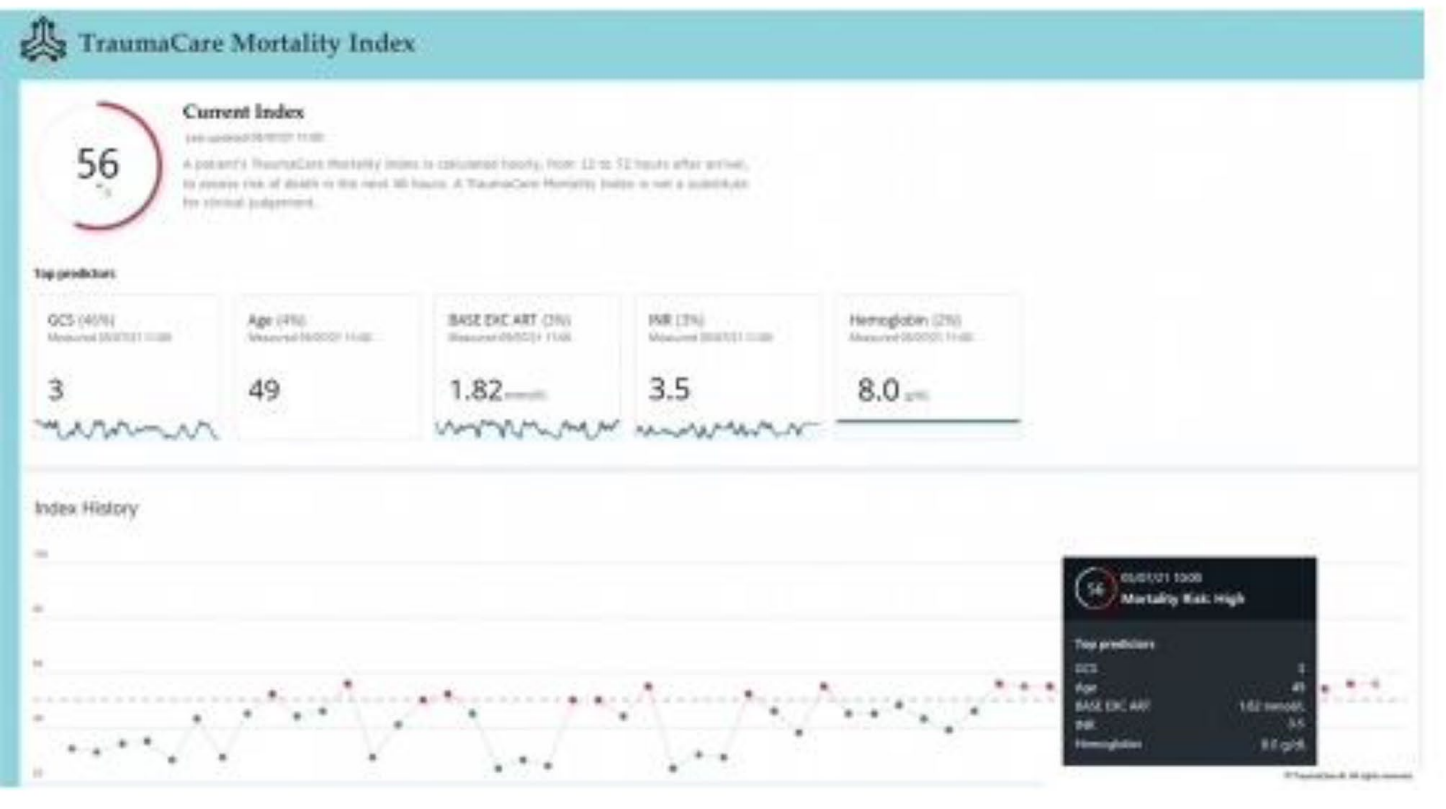
Parkland trauma index: The index determines the risk of mortality online and is underlying in the hospital documentation system

Figure 3 demonstrates a clinical scenario determined in the Parkland trauma index. It determines the risk of mortality online and is underlying in the hospital documentation system. Acute patient data are thus collected to determine issues of in house mortality [47].

## Discussion

Prior to the availability of digital processing of data, clinicians usually reported on a rather small number of patient and almost all of them summarized personal experience, or an evidence level up to level IV. Along with the availability of digital data and their processing, data generated in the local hospital setting were usually documented in local databases. Among the first attempts to collect data across hospitals was the Major Trauma Outcome Study (MTOS), which collected data from multiple regions in the USA. The documentation was collected by study nurses and other documentation experts [48].

Subsequently, digital documentation improved the options to process information across hospitals and was pursued on a regular basis. Trauma registries used the same principles, as seen in Great Britain in the development of a nationwide database, the Trauma audit and research network (TARN). Within Europe, the largest was the German Trauma Registry, which was the first to document beyond borders, mostly within European countries [49].

One of the drawbacks of Trauma registries has been the availability of data that require information beyond the data sets obtained. In addition, the quality control is usually not available until all information has been obtained from every single hospital, which takes usually about a year after collection of the data. Therefore, any additional research questions that went beyond these data sets could not be answered.

In trauma and orthopaedic care for acute major fractures, surgical decision making is an important factor to ensure an uneventful hospital course. This holds especially true for the transition of principles to stabilize major fractures, which may serve as a good example. The concepts moved from an “Early Total Care versus Damage Control Orthopaedics” discussion towards “Early appropriate care”, followed by “Safe definitive Surgery” (Table 3). The development of these concepts has been made available by specialized documentation of parameters used to determine the effect of surgery on parameters indicative of the clinical course. These parameters covered several pathogenetic cycles, namely “shock”, “coagulopathy”, “hypothermia”, and parameters indicative of “severe soft tissue injury”.

The belief was that inflammatory changes are set at the time of injury and can be influenced if another infect of clinical impact was too strong (second hit theory). Subsequently, the genetic storm theory hypothesized that the initial impact of trauma sets up the patient for any possible complications and the influence of therapy was doubted [50]. Nevertheless, later it became obvious that numerous secondary effects are certainly able to influence the further course, i.e. by infectious stimuli (PAMPS).

The development of these different concepts went along with improved evidence levels, i.e. from level IV to level II. The data warehouse concept (Table 2), although based on a single center only, used deductive information from a hospital database that covers all laboratory parameters and clinical data [51].

On a different level, prediction of complications has been a very important issue. In early attempts, expert opinion has been used to specify diagnoses. Among the most important ones relevant for an uneventful hospital course has been the consensus for pulmonary failure, i.e. adult respiratory distress syndrome, which lead to an ARDS consensus definition. In trauma patients, the severity of chest trauma can be crucial to determine the given risk for pulmonary complications. Although based on a local database, the development of the Thoracic Trauma Score (TTS) has been proven to have a predictive ability towards the development of ARDS [41]. These scorings required the availability of certain isolated factors but did not respect the time dependent changes of serial parameters. This can be overcome by having direct access to hospital data, which is currently only achieved by 2 different projects. Both have the privilege of using direct access to hospital data.

The WATSON visualization tool has been developed by IBM and was thought to utilize data in order to visualize possible upcoming complications during the hospital course. It is accepted as a teaching tool for residents in training and offers both, the use of a set database, and the utilization of the hospital system in the background [11]. The visualization originally derives from the development of turbines, which was invented in order to document how different shapes may lead to different types of thrust and outflow of air. From that, it has been modified to cover the course of patients for different indications. The implementation of the Trauma tool was covered in a time course between 2018 and 2022, with the first 2 year serving for development, followed by application within the hospital system and use for teaching and research [2]. It covers two different layers of information. At first, the true data of a patient are included and cover age, injury severity and other basic laboratory values (Fig. 2).

The tool developed in Dallas is named the Parkland Trauma Index of Mortality (PTIM) [21] and was installed around the same time and also uses a hospital information system (EPIC). The PTIM is a machine learning algorithm using emergency room data to predict mortality within 48 h in trauma patients during the first 3 days of their hospitalization. As a novel feature, the tool is integrated directly into the electronic health record of the hospital, extracts the data (i.e. 23 parameters) automatically, and calculates the PTIM score, thus requiring no input from the clinician (Fig. 3). Similar to the WATSON visualization tool, the PTIM may be used in the future to guide decision-making for important treatment strategies.

One may argue that the large data sets created by registries provide sustained evidence, as outlined by their country wide distributions. In contrast, they are also associated with well described drawbacks. As deductive data sets, their content has usually been consented by an expert group prior to testing and thus may have been subject to limitations. Moreover, the number of parameters is usually focused on basic information to describe patients – and their injury distribution and injury severity – rather than focusing on physiology. If physiological data are documented, the clinical parameters highlight those indicative of haemorrhage and cardiovascular parameters, maybe oxygenation, but these are usually limited to the admission period, thus precluding from making meaningful conclusions regarding the effect of treatment [52]. In addition, any cofactors that may affect outcome, are not available. These may include comorbidities that go beyond Diabetes mellitus and similar ones, or parameters descriptive of the clinical course. This is important, as parameters thought to be of sustained value for the severity of trauma and haemorrhage, such as lactate and pH, are modified by treatment within the first 24 h after admission. Dezman and colleagues were able to convincingly demonstrate that serial lactate levels outweigh the predictive ability of admission lactate values by far [53]. Furthermore, a connection between data regarding the severity of injury and clinical data is difficult to achieve. Thereby, if registries alone had been utilized to develop treatment concepts, these would have provided insufficient information.

In this line, it may be worth considering the importance of a local data distribution on a hospital level. These allow to collect a much more complex set of individual data that would include laboratory values and organ specific parameters. This approach has been used to test large local data sets, e.g. for development of scores, such as the Moore score for abdominal injuries, the Thoracic Trauma Score to evaluate chest injuries, and the Watson Analytics trauma tool (Table 2). The basis for being able to use a data set is the ability to extract data from a hospital system, as can be done using a data warehouse project (Fig. 1). This may allow for selecting clinically relevant data on admission, which may be extended throughout the hospital course. Local databases have been used to test the clinical relevance of subclinical parameters relevant for the risk of complications. These have helped understand how important the reactions of the inflammatory cascade are for the development of clinical complications. The prediction of SIRS, ARDS, and multiple organ failure was enabled by testing certain variables that had to be measured in specifically identified trauma patients [54] (Table 2).

### Limitations

We are aware that this review may not be complete and represents a selection that has a trauma specific background. It is evident that other subspecialties have generated larger registries, more focused and equally associated with certification processes (e.g. certain oncologic diseases, ICU data regarding sepsis etc.). Among the surgical subspecialties, our review may be limited because it deals predominantly with a more orthopaedic background, rather than focusing on truncal injuries. Moreover, its span may be limited due to the fact that search terms for this particular topic are not readily available. Finally, the focus of our review may have been based on the experience of the authors. However, since all they have contributed to the topic in various ways, we feel that this view still may represent many general trends in the reaction to the availability of big data.

## Conclusions

The development of several online tools to assess patients in a parallel fashion may suggest that the development of acute data acquisition will be helpful in the management of patients with complex, and rapidly changing, clinical situations. The development of a safe definitive surgery concept and its inclusion of multiple pathogenetic pathways can be regarded as a mode of development into flexible decision making, when compared with previous dichotomic approaches.

In addition, the development of AI has made a vast progress. While the projects listed above, PTIM and WATSON, both represent general AI, further steps are to be expected. It is possible that fracture recognition will belong to the easiest achievements of AI in the near future. In addition, machine learning might outgrow the current options of outcome prediction. It will be interesting to see how fast the changes will occur.

## Data Availability

No new data were created or analyzed in this study. Data sharing is not applicable to this article.
